# Patterns of Assemblage Structure Indicate a Broader Conservation Potential of Focal Amphibians for Pond Management

**DOI:** 10.1371/journal.pone.0160012

**Published:** 2016-07-26

**Authors:** Elin Soomets, Riinu Rannap, Asko Lõhmus

**Affiliations:** Institute of Ecology and Earth Sciences, University of Tartu, Tartu, Estonia; University of Sao Paulo, BRAZIL

## Abstract

Small freshwater ponds host diverse and vulnerable biotic assemblages but relatively few conspicuous, specially protected taxa. In Europe, the amphibians *Triturus cristatus* and *Pelobates fuscus* are among a few species whose populations have been successfully restored using pond restoration and management activities at the landscape scale. In this study, we explored whether the ponds constructed for those two target species have wider conservation significance, particularly for other species of conservation concern. We recorded the occurrence of amphibians and selected aquatic macro-invertebrates (dragonflies; damselflies; diving beetles; water scavenger beetles) in 66 ponds specially constructed for amphibians (up to 8 years post construction) and, for comparison, in 100 man-made ponds (created by local people for cattle or garden watering, peat excavation, etc.) and 65 natural ponds in Estonia. We analysed nestedness of the species assemblages and its dependence on the environment, and described the co-occurrence patterns between the target amphibians and other aquatic species. The assemblages in all ponds were significantly nested, but the environmental determinants of nestedness and co-occurrence of particular species differed among pond types. Constructed ponds were most species-rich irrespective of the presence of the target species; however, *T*. *cristatus* was frequent in those ponds and rare elsewhere, and it showed nested patterns in every type of pond. We thus conclude that pond construction for the protected amphibians can serve broader habitat conservation aims in the short term. However, the heterogeneity and inconsistent presence of species of conservation concern observed in other types of ponds implies that long-term perspectives on pond management require more explicit consideration of different habitat and biodiversity values. We also highlight nestedness analysis as a tool that can be used for the practical task of selecting focal species for habitat conservation.

## Introduction

Freshwater ecosystems have rich and unique biodiversity, which is under severe threat throughout the world due to overexploitation, pollution, hydrological modification, habitat degradation, and invasion by exotic species [1]. This biodiversity crisis is widely recognised and a lot of resources have been allocated to the conservation of lakes and rivers over the last 30–40 years [2]. Much less attention has been paid to conserving the biodiversity of small lentic freshwater bodies, such as ponds, natural depressions, floods and vernal pools [3, 4]. Yet, these small freshwater bodies are very valuable habitats, which, by supporting many unique and rare species, play a central role in maintaining high regional biodiversity [5, 6]. In many regions, small freshwater bodies are under serious anthropogenic threat due to changed land use and agricultural intensification [6–8], notably draining, pollution, eutrophication, fish stocking, and mismanagement [9, 10].

It is well known that anthropogenic pressures on small freshwater bodies can be mitigated by pond management for biodiversity, and many small-scale actions have been implemented [11]. There are, however, at least three major concerns that urge conservationists to take more systematic and broader-scale approaches. First, the naturally fragmented pond habitat implies metapopulation dynamics to be common in aquatic and semi-aquatic freshwater species (e.g., [12, 13]). Such populations depend on the maintenance or restoration of habitat connectivity in addition to habitat quality at the pond scale. For example, Semlitsch and Bodie [5] demonstrated how the viability of amphibian populations can be lost due to increasingly impaired dispersal within a shrinking pond network. Secondly, while a (meta-) population approach can be implemented for conspicuous taxa (such as some amphibians), the ultimate aim should be to preserve the whole pond biodiversity, most of which is difficult to monitor comprehensively. Hence, there is a need for practical analyses of species co-occurrence patterns in ponds, to understand how well, and where, conspicuous species represent other taxa (e.g.,[14, 15]). Thirdly, to address different threats and all parts of the pond biodiversity in a cost-effective way, a systematically selected set of ‘focal species’ (sensu [16]) may be needed.

According to Lambeck [16], a set of focal species should comprise the most threat-sensitive and/or rarest species representing four main categories: area -, resource -, process—and dispersal-limited species. This approach first requires the identification of processes that threaten biodiversity [17] and it should be separately demonstrated that the focal species co-occur with other species of conservation concern [18, 19]. So far, there exists no established protocol for selecting focal species for biodiversity conservation [20, 21] and practical problems abound (e.g., insufficient data quality; limited numbers of potential focal species; researcher’s failure to comprehensively identify limiting factors; [22]). Perhaps most importantly, however, the focal species approach will remain an academic exercise unless the habitat is (successfully) managed in practice. Therefore, the existing, publicly accepted habitat conservation activities for charismatic species constitute an important system for effectiveness research [23].

In this paper we report on the potential of protected amphibians as focal species for pond biodiversity. So far, there are only few cases where pond restorations has helped threatened amphibians [24–26], although common species have thrived [27–29]. It is known that pond management can also support some less conspicuous species, notably aquatic macro-invertebrates [11, 30–32], but, again, only a few success stories exist for threatened taxa [33, 34]. Thus, the main failure of pond management has been its low value for threatened species and it is important to further explore wider benefits of the few success stories.

Our study is built up on an example of a large-scale pond restoration and construction project that was successful for two threatened European amphibian species, the northern crested newt (*Triturus cristatus*) and the common spadefoot toad (*Pelobates fuscus*), in Estonia [25]. The populations of these European species have an overall decreasing trend [35, 36] and they are thus strictly protected throughout the European Union (Annexes II and/or IV of the Habitats Directive, 92/43/EEC) [37]. We first analyse the assemblage structure (nestedness of amphibian and macro-invertebrate assemblages) comparatively in the ponds specifically constructed for the two amphibians (hereafter: target species), and in man-made and natural ponds, and we identify some habitat factors shaping the nestedness patterns. The comparison among pond types was aimed to assess the relative value of constructed ponds for the target species and other threatened species (note that pond construction is rather costly), which also reflects their habitat sensitivity. We then specifically explore the co-occurrence of the target species with the other taxa to assess their potential for a focal species scheme. We use the formal nestedness analysis as the central tool (e.g., [38]) and, thus, our analysis also contributes to a more basic understanding of nestedness patterns among amphibians and aquatic macro-invertebrates in different types of small freshwater bodies (see also [39–42]).

## Material and Methods

### Study area

**Fig 1:**
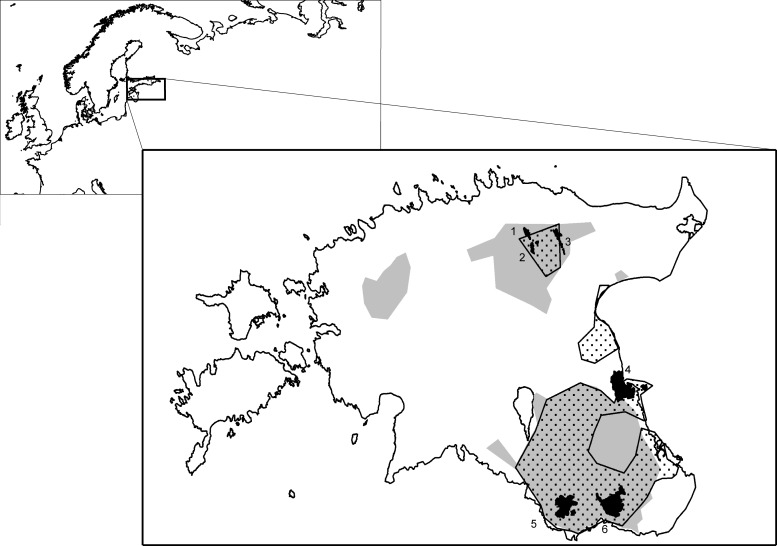
**The location of study sites (black areas) in Estonia:** 1 –Neeruti reserve; 2 –Porkuni reserve; 3 –Mõdriku-Roela reserve; 4 –Emajõe-Suursoo NPA; 5 –Karula NP; 6 –Haanja LPA. The grey area and the dotted area show the distribution area of *T*. *cristatus* and *P*. *fuscus*, respectively; as revealed by country-wide systematic surveys of small water-bodies in 2007–2015.

Our study was conducted in northern, eastern and southern Estonia, following the national distribution area of *Triturus cristatus* and *Pelobates fuscus* (Fig 1). Estonia is situated in the hemiboreal vegetation zone; the mean January and July temperatures across the study area are –6°C and 17°C, respectively. We sampled six protected areas where large-scale pond construction (restoration of overgrown and silted ponds or creation of new ones) has been carried out specifically for *T*. *cristatus* and/or *P*. *fuscus* since 2004 (Fig 1). For a comparison with 66 such ponds specially constructed for amphibians in 2005–2011, we also explored 65 natural ponds (e.g., in depressions, beaver floods, small karst lakes) and 100 man-made ponds created for cattle or garden watering, peat excavation, fish cultivation or for sauna use. These proportions roughly correspond to the actual ratio of the pond types in the study area. The pond sizes ranged from 0.003 ha to 6.72 ha (mean 0.5 ha).

Two of the study areas, Karula National Park (NP) (26°29’ E; 57°42’ N) and Haanja Landscape Protected Area (LPA) (27°2’ E; 57°43’ N), are situated in the hilly, well-forested southern Estonia with small scattered settlements and agricultural lands. Numerous lakes, beaver floods, swamps and human-created ponds are found in these reserves. Emajõe-Suursoo Nature Protected area (NPA) (27° 12’E; 58° 22’N) is located in eastern Estonia and encompasses one of the oldest and largest Estonian delta swamps (20,000 ha), at the mouth of the Emajõgi River. The lentic water-bodies include flooded marshes and lakes. Small ponds in the agricultural areas can be found on sandy ridges bordering the nature reserve. We also sampled three smaller reserves–Mõdriku-Roela (1621 ha), Neeruti (1271 ha) and Porkuni (1145 ha)–in northern Estonia on the Pandivere Upland (26° 18’ E, 59°10’ N; 2415 km^2^ large), which is the highest bedrock upland in Estonia. The terrain is hilly moraine with clearly defined eskers that rise over 160 m a.s.l. Agricultural landscapes dominate the area; grasslands and conifer-dominated forests cover ca. 30%. The raised limestone topography causes intense filtration and karst processes, such that many temporary lakes are formed during the snowmelt and rainfall in the spring, but they typically dry out by late summer–early autumn.

In Estonia the Environmental Board is a manager of all protected areas. It is a public body which falls within the area of governance of the Ministry of the Environment. The Environmental Board is also responsible for the organisation of monitoring activities in protected areas, and our study was part of it. All the study sites and activities, as well as field methods were agreed with the Environmental Board before the outset of the fieldwork, thus, specific permissions were not needed.

### Fieldwork

We searched for all the eight amphibian species that have been recorded in the study areas: *Lissotriton vulgaris*, *Triturus cristatus*, *Bufo bufo*, *Pelobates fuscus*, *Pelophylax lessonae*, *P*. *esculentus*, *Rana arvalis and R*. *temporaria*. In addition to *T*. *cristatus and P*. *fuscus*, also *P*. *lessonae* and *R*. *arvalis* are included in the EU Habitats Directive (Annex IV). Among the diverse macro-invertebrate fauna of small freshwater bodies [43], we included all odonates (Odonata: Anisoptera; Zygoptera) and a selection of large water beetles (Coleoptera: Dytiscidae; Hydrophilidae). The insect sets included three species of dragonflies (*Leucorrhinia albifrons*, *L*. *pectoralis* and *L*. *caudalis*) and two water beetles (*Dytiscus latissimus* and *Graphoderus bilineatus*) that are strictly protected by the EU Habitats Directive. The two latter beetle species also have a regionally vulnerable status ([44]; Table 1).

**Table 1:** Pond type-specific frequencies of occurrence of amphibians and aquatic insects, and the NODF-based significance of fit with a nested assemblage pattern in protected species (listed in the Annexes II and IV of the EU Habitats Directive).

	Frequency of occurrence (%)					** **
	and fit with the nested pattern (p)					
			
	Constructed ponds		Natural ponds		Man-made ponds	
	(N = 66; 24 taxa)		(N = 65; 31 taxa)		(N = 100; 31 taxa)	
	%	p	%	p	%	p
**Amphibians**						
*Pelobates fuscus*	25	0.132	17	0.055	7	0.091
*Triturus cristatus*	60	0.001	22	0.001	10	0.022
*Bufo bufo*	9		31		34	
*Lissotriton vulgaris*	95		51		56	
*Rana arvalis*	15	0.151	45	0.001	26	0.001
*Pelophylax sp*.	86		46		68	
*Rana temporaria*	34		34		22	
**Dragonflies and damselflies**						
*Coenagrion armatum*	0		3		0	
*C*. *hastulatum*	7		20		27	
*C*. *puella*	7		18		30	
*C*. *pulchellum*	6		12		11	
*Cordulia aenea*	63		18		23	
*Enallagma cyathigerum*	1		0		5	
*Epitheca bimaculata*	4		6		0	
*Erythromma najas*	0		9		14	
*Leucorrhinia albifrons*	13	0.463	5	0.277	4	0.001
*L*. *caudalis*	4	0.455	17	0.346	0	
*L*. *dubia*	3		3		2	
*L*. *pectoralis*	48	0.084	34	0.091	13	0.024
*L*. *rubicunda*	33		14		9	
*Libellula depressa*	0		0		5	
*L*. *quadrimaculata*	81		17		30	
*Orthetrum cancellatum*	0		0		5	
**Diving beetles and water scavenger beetles**						
*Acilius canaliculatus*	7		3		2	
*A*. *sulcatus*	68		11		6	
*Cybister lateralimarginalis*	0		9		3	
*Dytiscus latissimus*	0		3	0.264	1	0.001
*D*. *marginaalis*	0		2		4	
*Graphoderus bilineatus*	0		18	0.148	3	0.001
*G*. *cinereus*	12		17		8	
*G*. *zonatus*	18		0		0	
*Hydaticus seminiger*	0		5		2	
*H*. *transversalis*	0		9		6	
*Hydrochara caraboides*	0		6		2	
*Hydrophilus aterrimus*	3		5		5	

Field data were collected in June either in 2010, 2011 or 2013 during the larval period of the two target amphibians. Each pond was visited once during the whole study period, and dip-netted by one experienced herpetologist and one experienced entomologist who also recorded the area of water body and shade from the surrounding trees (% of the water table). We used a standard dip-netting of amphibian larvae [45] with a hand dip-net (40 x 40 cm frame) as the main method for detecting amphibians (presence of larvae). We swept the dip-net through different water layers, covering all important microhabitats for amphibians. This method is highly effective to detect amphibians’ larvae, as demonstrated earlier [46]. The dip-netting time varied between water bodies: we dip-netted up to 45 minutes and sampling effort increased along with the pond size. The absence of species was only concluded after 45 minutes of dip-netting. In addition, eggs of newts and egg-clusters of the “green frogs” (*Pelophylax lessonae/esculentus*) were searched for. The latter were treated collectively, given the difficulties in distinguishing the eggs and tadpoles of the pool frog (*P*. *lessonae*) and the edible frog (*P*. *esculentus*). Odonate larvae and water beetles (both adults and larvae) were actively searched for at each site by sweeping a hand dip-net (40 x 40 cm frame) through the vegetation and detritus material. This survey method has proven to be the most time-effective [47].

We established the presence of fish, using combined data of dip-netting (described below), visual observation and information from local people (S1 Table). As the fieldwork was carried out by experienced herpetologists and entomologists, all caught specimens were detected in the field and thereafter released into their natal ponds.

### Analytical procedures

Our framework refers to the focal species approach, which involves identification of a suite of species targeted to habitat management, each focal species acting as a surrogate for other species [16]. We followed three steps (criteria) for assessing our target species within this framework. First, since the focal species approach relies in part of nestedness pattern among assemblage [18], we established both assemblage- and species-scale nestedness patterns in each type of pond [48]. Secondly, we considered the fact that lack of presence data and habitat suitability may be a problem of focal species selection (see also [18]). Thus, we studied *P*. *fuscus* and *T*. *cristatus* abundance in each type of pond. Thirdly, we interpreted the habitat-sensitivity of the potential focal species distribution as a combination of relatively uncommon occurrence (e.g., the < 25% frequency criterion [48, 49]) and its difference between specially constructed and other ponds.

Our main nestedness analyses were based on the relatively conservative NODF metric (Nestedness based on Overlap and Decreasing Fill), which ranges between 0% (maximal scatter) and 100% (perfectly nested). NODF is based on standardized differences between rows (sites) and columns (species) and the paired matching of occurrences in this matrix, so that the value does not depend on the matrix shape, size, or column-row transition, and one can disentangle nestedness patterns for particular species or types of sites [50, 51]. We used the ANINHADO software [52] for analysing both the full presence/absence matrix (water bodies ordered by species presence, i.e., most presences in the left-top corner of the matrix) and, separately, for sub-matrices for each of the three pond types and for each protected species found (S2 Table) Statistical significance of nestedness was estimated by comparing the observed NODF value with that of 1000 permuted matrices where the presences were randomly assigned among cells (‘ER model’; [50, 53]).

To specifically assess *T*. *cristatus* and *P*. *fuscus* as indicators for other species of amphibians and aquatic macro-invertebrates listed in the EU Habitats Directive (see above), we used two approaches. First, we used Generalized Linear Models (GLM; based on Poisson error distribution and log link function) for analysing the number of accompanying species in relation to the pond type and incidence of each target species at the time of the sampling. These analyses also included an interaction term. Secondly, we elaborated the nestedness analyses at the scale of individual species-pairs. We used the software ANINHADO for ordered-by-species-presence matrices [52, 54] to calculate paired overlap (PO_ij_) between the presence of each target species with that of other amphibians and aquatic macro-invertebrates [50, 55]. For columns (species), PO_ij_ is the share of presences in a given column *j* (here: other amphibian and aquatic macro-invertebrate species) that are located at identical row position to those in column *i* (target species). Statistical significance of PO_ij_ was assessed by comparing the observed PO_ij_ value with 1000 permuted PO_ij_ value produced by ER null model algorithms [50, 52, 53].

To test for differences in the presence of fish between specially created ponds and other pond types, we used χ^2^-test. For determining the impact of environmental factors on the assemblage nestedness, we used Lomolino’s “departures method” [56]. The principle is to quantify unexpected presences of species (‘departures’) for alternative matrix configurations that order the sites along with the environmental gradients (S3, S4 and S5 Tables). We ordered water bodies according to species occurrences and (i) total area, (ii) shade and (iii) age in specially created ponds, and compared those patterns with the randomized main matrix and the pond-type sub-matrixes (see above). We also studied the nestedness pattern according to the presence and absence of fish. We divided the dataset of each pond type into two groups–ponds with fish and without fish. We used the NestSim software to estimate the departures and to calculate the percent perfect nestedness (%PN): %PN = 100 * ((R-D)/R); where R is the mean number of departures sorted by column; and D is observed numbers of departures sorted by column. We used the outcomes of each sorting against 1000 random permutations to determine the p-value for each factor.

## Results

### Faunal richness of the ponds

We recorded a total of seven species of amphibians, 16 species of odonates, and 12 species of water beetles (from the pre-defined list) in the 231 water bodies (Table 1, S2 Table). Only three water bodies lacked any of those species. In 159 ponds, at least one species protected by the EU Habitats Directive was found (maximum: five species; eight in total): in 61 (92%) constructed ponds, in 48 (48%) man-made ponds, and in 50 (77%) natural ponds. *Leucorrhinia pectoralis*, *Triturus vulgaris* and *Pelobates fuscus* were the most frequent protected species, while *Dytiscus latissimus* was the rarest. The presence of fish was a significantly less frequent in constructed ponds (7.7%) than natural ponds (43.9%; *χ*^*2*^ = 21.42; p < 0.0001) or man-made ponds (61.1%; *χ*^*2*^ = 45.39; p < 0.0001).

**Fig 2:**
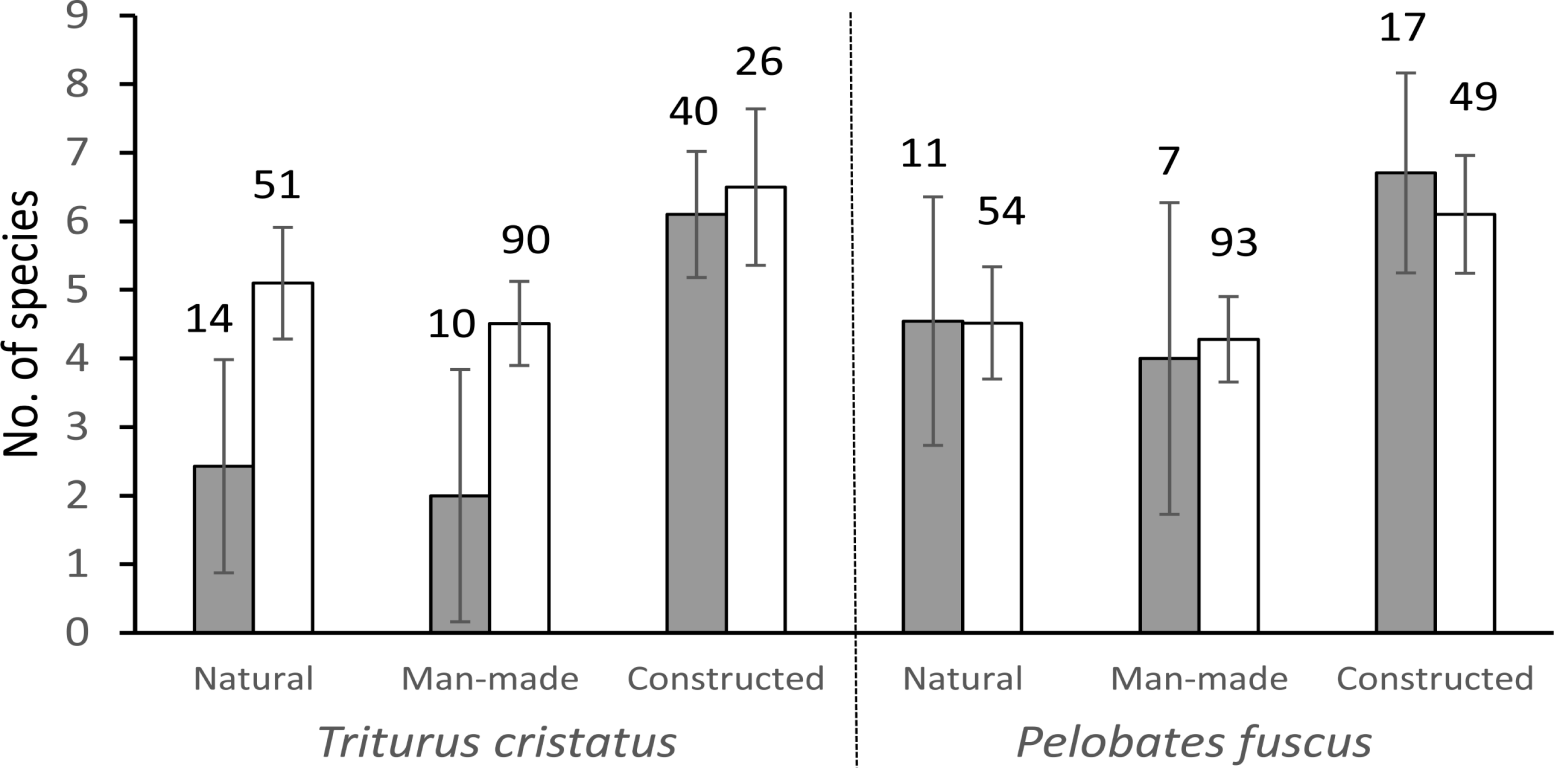
The number of studied species in ponds with and without focal species. Filled bars are ponds of focal species presence (*T*. *cristatus* or *P*. *fuscus*) and empty bars are ponds of focal species absence; whiskers are 95% confidence intervals; number above the bars represent the N–value of each type of pond with and without focal species.

The mean number of recorded amphibian and macro-invertebrate species across all studied ponds was 5.3 ±3.2 (SD) but that varied significantly among pond types (GLM, likelihood-ratio test: χ^2^_2_ = 54.3, p < 0.001). The largest difference was between species-rich constructed ponds (7.1 ±2.2 species) and both the natural (4.9 ±3.7) and man-made ponds (4.4 ±3.0), but all the contrasts were highly significant (p < 0.001). Similar pond-type influences persisted in the analyses on the species accompanying the target species of pond construction, *T*. *cristatus* and *P*. *fuscus* (Fig 2). Thus, in the case of *P*. *fuscus*, the number of accompanying species (excluding both target species) only depended on the pond type. In contrast, *T*. *cristatus* had an independent effect, which, additionally, depended on the pond type (Table 2). The interaction indicated that presence of *T*. *cristatus* had no effect on the accompanying species in the constructed ponds, while it negatively affected them in natural and man-made ponds (Fig 2).

**Table 2:** The effects of pond type (3 types) and the presence of target species (*T*. *cristatus* or *P*. *fuscus*) to the number of other considered amphibian and insect species. The effects refer to likelihood-ratio tests for Generalized Linear Models based on Poisson error distribution and log link function.

	*T*. *cristatus*		*P*. *fuscus*	
Factor (df)	χ^2^	p	χ^2^	p
Pond type (2)	63.0	< 0.001	22.6	< 0.001
Target species (1)	32.9	< 0.001	< 0.1	0.905
Pond type × Target species (2)	18.0	< 0.001	0.6	0.740

### Nestedness patterns

The studied assemblages were significantly nested both at the scale of pond types and overall (Table 3; S2 Table). The nestedness values were larger for sites (NODF_row_) than for species (NODF_column_); that contrast was particularly clear (and significant) in constructed ponds. Among the eight protected species, only *T*. *cristatus* fitted with a nested model in every pond type; in *Rana arvalis*, *Graphoderus bilineatus* and *D*. *latissimus* such fit was observed in two pond types. The protected odonates showed a nested pattern in man-made ponds only. For *P*. *fuscus* the null-hypothesis of random assembly could not be rejected at all, at α = 0.05 (Table 1).

**Table 3:** Nestedness of the aquatic assemblages by pond type, according to the NODF statistic. NODF metric (Nestedness based on Overlap and Decreasing Fill) ranges between 0% (maximal scatter) and 100% (perfectly nested).

		NODF values					Significance	
			Simulated					
Pond type	NODF	Observed	Mean	Min.	Max.	SD	Z	p
Constructed	Total	53.7	21.7	18.6	25.2	1.1	29.6	<0.001
	Species	22.0	21.4	17.7	25.0	1.1	0.5	0.302
	Sites	62.4	21.8	18.4	25.3	1.1	37.3	<0.001
Natural	Total	33.5	15.8	15.9	23.0	1.0	18.5	<0.001
	Species	27.4	19.2	15.3	23.1	1.0	8.0	<0.001
	Sites	35.3	19.2	16.1	22.8	1.0	16.6	<0.001
Man-made	Total	42.1	14.0	11.7	16.1	0.8	37.5	<0.001
	Species	27.0	13.4	11.1	15.6	0.8	16.7	<0.001
	Sites	44.2	14.0	11.7	16.1	0.8	40.2	<0.001
All ponds pooled	Total	43.7	16.6	15.2	18.1	0.5	55.3	<0.001
	Species	32.8	15.8	14.2	17.5	0.6	30.9	<0.001
	Sites	44.0	16.7	15.2	18.1	0.5	55.7	<0.001

The presence of fish impacted the nestedness patterns in different pond types. In constructed ponds without fish the species assemblages were significantly more nested (NODF = 51.86; p < 0.01; N = 60) than in ponds with fish (NODF = 13.14; p = 0.93; N = 5). Contrary, in natural and man-made ponds the species assemblages were less structured in ponds which did not consist fish (natural ponds: NODF = 26.14; p < 0.01; N = 32; man-made ponds: NODF = 28.1; p < 0.01; N = 35) than in ponds with fish (natural ponds: NODF = 34.75; p < 0.01; N = 57; man-made ponds: NODF = 42.04; p < 0.01; N = 55).

Both pond size, shade, and age supported the formation of the nested assemblage pattern and there were apparent differences among pond types. Thus, pond size was a significant factor for man-made ponds only, while shade (and not size) affected natural and constructed ponds (Table 4; S3 and S4 Tables). The age of the pond was only known for the constructed ponds (Table 4; S5 Table).

**Table 4:** Influence of shade, pond size, pond age on the assemblage nestedness by pond type. Percent perfect nestedness and its significance have been calculated according to Lomolino [56]. Sample sizes vary slightly depending on the availability of the environmental data.

		Pond type		
Characteristic	Natural	Constructed	Man-made	Total
**Shade**				
No. of sites	51	62	90	203
Mean shade of sites (%)	22	10	16	15.7
Min. shade (%)	0	0	0	0
Max. shade (%)	100	100	100	100
% perfect nestedness	8.5	15.5	4.4	18.2
p-value	0.02	<0.001	0.08	<0.001
**Pond size**				
No. of sites	62	66	98	226
Mean area of sites (ha)	1.31	0.10	0.29	0.51
Min. area of sites (ha)	0.003	0.01	0.01	0.003
Max. area of sites (ha)	6.72	3.00	5.20	6.72
% perfect nestedness	1.9	-1.5	6.3	8.4
p-value	0.31	0.68	0.02	<0.001
**Pond age**				
No. of sites	-	59	-	-
Mean age of ponds (y)	-	7.68	-	-
Min. age of ponds (y)	-	3	-	-
Max. age of ponds (y)	-	9	-	-
% perfect nestedness	-	17	-	-
p-value	-	<0.001	-	-

## Discussion

### Pond construction for protected amphibians

Our analyses confirmed a relatively high occupancy of specially constructed ponds not only by the two target species, *Triturus cristatus* and *Pelobates fuscus*, but also by other amphibians and aquatic insects including several other protected taxa. These ponds were specially constructed for *T*. *cristatus* and/or *P*. *fuscus*, taking their habitat demands into account. The constructed ponds had gentle slopes and rather large shallow littoral zone, the maximum depth of water varied from 0.4m to 2.5 m [25]. None of the constructed ponds was allowed a connection to running water (ditch, stream, river) to avoid fish introduction or sedimentation. It could be the reason why fish was rarely present in constructed ponds compare to the natural ponds. Land cover within 50 m from any constructed pond was mainly to consist of a mosaic of forest and (semi)natural grassland (for *T*. *cristatus*) and (semi)natural grasslands and small extensively used potato fields or vegetable gardens (for *P*. *fuscus*) [25], while natural ponds were often surrounded by forest [57]. Previous studies have demonstrated that pond construction may support assemblage richness comparable to natural wetlands [30, 58] but, in our study, specially constructed ponds even outperformed natural ponds. We recall, however, that a seemingly low quality of natural habitat may be, at least in some species, a sampling artifact since we only sampled the natural water-bodies that were comparable with man-made ponds in terms of size and water conditions. For example, both diving beetles of conservation concern, *Dytiscus latissimus* and *Graphoderus bilineatus*, have their main populations in lakes [59, 60].

Another key finding was that the time since pond construction affected the nestedness of the pond fauna. Considering also the distinct contrast between site- and species-scale nestedness in this pond type (Table 1), the time effect probably reflects the rapid, but initially stochastic, processes of habitat development and species colonization in the taxa studied (e.g., [61–63]). Such parallel development could explain, for example, why significant co-occurrences of odonate species and the two target amphibians were mostly observed among the constructed ponds (Table 5).

**Table 5:** Species-pair level co-occurrence (paired overlap, PO_ij_) of the target species and other species of amphibians and macro-invertebrates by pond type. PO_ij_ has been calculated only for the cases with at least three records of both the target and the other species; an asterisk (*) indicates non-random co-occurrence at p < 0.05.

	Target species, pond type, and PO_ij_ (%)					
Accompanying species	*Triturus cristatus*			*Pelobates fuscus*		
	Constructed ponds	Natural ponds	Man-made ponds	Constructed ponds	Natural ponds	Man-made ponds
**Amphibians**						
*T*. *cristatus*				47	45*	14*
*P*. *fuscus*	47	45*	14*			
*B*. *bufo*	50*	7	20*	17*	9*	0
*L*. *vulgaris*	100	71*	80*	88*	72*	71*
*P*. *lessonae/esculentus*	87	7	10*	94*	45*	86*
*R*. *arvalis*	50*	50*	40*	30*	36*	29*
*R*. *temporaria*	52	21	20*	47*	9	29*
**Dragonflies and damselflies**					
*C*. *hastulatum*				80*	18*	29*
*C*.*puella*				80*	18*	43*
*C*. *pulchellum*				75*	25*	0
*C*. *aenea*	65	0	0	65*	0	14*
*E*. *bimaculata*	33	0	0	33*	0	0
*L*. *albifrons*	56*	0	0	22*	0	0
*L*. *caudalis*	33*	0				
*L*. *pectoralis*	63	7	10*	59*	27*	14*
*L*. *rubicunda*	59*	0	0	35*	10	0
*L*. *quadrimaculata*	88	0	10	0	9	43*
**Water beetles**						
*A*. *canaliculatus*				20*	50*	0
*A*. *sulcatus*	68	14*	17*	53	29*	50*
*C*. *lateralimarginalis*					17*	0
*G*. *cinereus*	63*	8	0	38*	36*	0
*G*. *zonatus*	75*			25*		
*H*. *seminiger*		50*			31*	
*H*. *transversalis*		17*	0		67*	0
*H*. *caraboides*		25*			75*	
*H*. *aterrimus*		67*	0		100*	0

These findings suggest that pond construction for *T*. *cristatus* and *P*. *fuscus* created quality habitats also for several other species of conservation concern and, thus, these two amphibians could be used as focal species for pond management. The keys for such success probably include: reversing the succession, consistent creation of limiting conditions (e.g., shallow-water areas and variability), and the elimination of fish [25]. In contrast, actual presence of the target species in those ponds did not add accompanying species in our study. Other types of ponds even had reduced number of accompanying species in the presence of *T*. *cristatus* (Fig 2; but see [64], for an opposite result). As *T*. *cristatus* is a predatory species [65], one reason can be its lasting predation influence on those longer-developed assemblages. This hypothesis deserves experimental study because it refers to a potential conservation dilemma–an unstudied issue in pond management.

### Nestedness patterns and the indicator value of amphibians

The general nestedness (NODF) values recorded in our study were moderate, 44–54 (cf. [50]), but their significance in each of the three pond types strengthens the evidence. Aquatic ecosystems often have loosely structured assemblages [41, 66, 67]. Therefore, the general nestedness condition of focal species selection [68, 69] was met in our pond study system. However, we also discovered that the nestedness was shaped by distinct environmental factors in different pond types. It therefore remains unanswered how the focal-species approach should be expanded beyond pond construction, i.e., in habitat management or priority site selection among existing natural and anthropogenic ponds.

The presence of fish impacted the nestedness structure differently in different pond types. In constructed ponds absence of fish impacted strongly the nestedness structure compared to the natural and man-made ponds where the nestedness pattern of species assemblages were structured by fish presence. Such contrary pattern in constructed versus natural and man-made ponds could be derived from the larger area of the latter pond types, as well as more extensive vegetation cover in natural ponds, creating larger variety of microhabitats. Similar impact of fish predation on the species patterns of wetland assemblages have also been demonstrated by Baber et al [70].

The man-made ponds were distinct for having pond area as a structuring factor (see also [40, 70]), even though natural ponds varied even more in size (Table 4). This may be related to some taxon-specific responses observed. For example, the conservation-concern *Leucorrhinia* species followed a nested pattern in man-made ponds only, and some amphibians (*Pelophylax sp*., *Bufo bufo*) were relatively frequent, while the target species were rare. These patterns refer to specific limitations (see also, e.g. [42, 71–73]) in man-made ponds, which were most permanent and most frequently hosted fish. The negative impact of fish could be mitigated by larger size of the ponds. Amphibians are sensitive to these habitat qualities, which probably caused their strong co-occurrence (Table 5). Therefore, conservation management for man-made ponds could prioritize their size-related habitat heterogeneity and amphibian species richness (rather than the single occurrences of the target species).

In natural ponds (also in constructed ponds), assemblage nestedness was affected by sun exposure (resp. shade) and these ponds had the most suitable conditions for vegetation development. Higher nestedness in more open water-bodies has been reported also in amphibians in Italy [74]. In temperate climate sun exposure creates areas with high water temperatures creating favorable habitat conditions for aquatic beetles [75–77] and promoting rapid development of amphibians’ and aquatic invertebrates’ larvae [78–81]. Possibly, this explains also the relatively large share of all records of water scavenger beetles in natural ponds and, specifically, their striking co-occurrence with amphibians (Table 5). Rich aquatic vegetation may explain, for example, the frequent occurrence of *Rana arvalis* and the generally rich dragonfly assemblages (cf. [38, 82]) in natural ponds. The fact that dragonfly distributions were almost entirely segregated from that of *T*. *cristatus* (Table 5) suggests complex predatory interactions worth of future study. In brief, the assemblage structuring processes in natural ponds might be different from those in recently constructed ponds, because natural ponds are often connected with running water, having higher possibility for fish colonization. They also have remained in forested landscapes while specially constructed ponds are situated mainly in mosaic landscapes. However, the differences of species assemblages in natural and specially created ponds may also derived from time of succession. The constructed ponds were in average 7 years old, which might not be enough for establishment of diverse macrophyte cover.

In general, following the steps of focal species selection and the results of analysis, we thus confirm that the assemblages were nested but, at the species level, *P*. *fuscus* showed no nestedness in any type of ponds (see Table 1). This species appeared too rare, which could be also partly related to more specific attention on *T*. *cristatus* in the pond construction. However, we found comparable numbers of *T*. *cristatus* and *P*. *fuscus* larvae from natural and man-made ponds, where *T*. *cristatus* still retained a more nested pattern. Furthermore, *T*. *cristatus* had 60% incidence in the constructed ponds, which were created following its requirements, but only 10% incidence in ordinary man-made ponds. Such difference confirms the habitat-sensitivity of this species and, considering also the persistent nestedness patterns, we can recommend it as an assemblage-scale focal species in constructed ponds as well. In contrast, *P*. *fuscus* did not meet these criteria in any type of pond.

Our study thus elaborates the discussion on the indicator value of amphibians for conservation purposes. Previously, it has been found that amphibians are probably poor cross-taxon indicators across landscapes involving both terrestrial and aquatic habitat (e.g. [20, 82]), but their value may be higher specifically for wetlands [83–85]. Our study demonstrates that the indicator value of amphibians differs among water-bodies in response to complex factors that affect species co-occurrence, where whole taxon groups (e.g., dragonflies and water scavenger beetles in our study) may have highly variable representation, depending on water-body type. We therefore advocate that, for pond management beyond the initial construction issues, the focal species should be selected from every major taxon group.

## Supporting Information

S1 Table Fish presence in different type of ponds. (XLSX)

S2 Table Main matrix with all types of ponds for nestedness analysis. (XLSX)

S3 Table The area of different pond types and species presence matrix for Lomolino's method. (XLSX)

S4 Table The shadiness of different pond types and species presence matrix for Lomolino's method. (XLSX)

S5 Table Matrix with species presence and age of constructed ponds for Lomolino's method. (XLSX)
