# Correction: Patterns of Assemblage Structure Indicate a Broader Conservation Potential of Focal Amphibians for Pond Management

**DOI:** 10.1371/journal.pone.0163007

**Published:** 2016-09-13

**Authors:** Elin Soomets, Riinu Rannap, Asko Lõhmus

A funder is missing from the Financial Disclosure of the published article. The full Financial Disclosure is: The study was financially supported by EU LIFE+ project LIFE08NAT/EE/000257 (DRAGONLIFE) and by the Estonian Research Council (grants no. 9051 and IUT 34–7).
